# Supplementary material for: From physical self-esteem to sports participation: The mediating role of exercise motivation and social support in adolescents
Source: PLoS One. 2025 May 22;20(5):e0324588. doi: 10.1371/journal.pone.0324588 (PMC12097568; doi:10.1371/journal.pone.0324588)
Supplement: S1 Data — (ZIP) [file pone.0324588.s001.zip › S1 Empirical data and original scale/S1 original scale.pdf]

From Physical Self-Esteem to Sports Participation: The Mediating Role of Exercise Motivation and Social Support in Adolescents

Dear Student,

Hello! Thank you for taking the time to participate in this survey. This questionnaire is designed to explore the relationship between physical self-esteem and sports participation in adolescents, with a particular focus on the mediating roles of exercise motivation and social support. The objective of this study is to understand the mechanisms through which these factors interact and to provide scientific evidence for strategies to promote adolescents' physical activity and overall well-being.

The questionnaire primarily consists of objective questions. Please answer based on your personal experiences. There are no right or wrong answers, as individual situations differ. Rest assured that all the information you provide will be kept strictly confidential, will not be shared publicly, and will not be used for any personal assessment. Your honesty is greatly appreciated, so please select the answers that best reflect your actual situation.

I hereby pledge to uphold the strictest confidentiality regarding all the data collected during this study.

Your participation is vital to the success of this research, and I sincerely thank you for your support and cooperation!

Part 1: Personal Information (Please mark "√" next to the appropriate option)

1. Gender: Male ( ) Female ( )

2. Grade:Middle school ( ) High school ( )

3. Residence type：Rural（ ） Urban（ ）

Part 2: Physical Exercise Level Scale

Please recall your physical exercise over the past month and select the corresponding answer (A, B, C, D, E) in the parentheses based on your actual situation.

1. **What type of physical exercise do you regularly engage in?** ( )

A. Light exercise (e.g., walking, doing light calisthenics)

B. Low-intensity, relaxed exercise (e.g., casual volleyball, table tennis, jogging, tai chi)

C. Moderate-intensity, more vigorous and enduring exercise (e.g., cycling, running)

D. High-intensity exercise with heavy breathing and sweating, but not very prolonged (e.g., badminton, basketball, tennis, soccer)

E. High-intensity and prolonged exercise with heavy breathing and sweating (e.g., racing, aerobic routines, swimming)

2. When engaging in the above intensity of physical activities, how many minutes do you usually spend each time? ( )

A. Less than 10 minutes

B. 11 to 20 minutes

C. 21 to 30 minutes

D. 31 to 59 minutes

E. 60 minutes or more

3. How many times per month do you engage in the above physical activities? ( )

A. Less than once per month

B. 2 to 3 times per month

C. 1 to 2 times per week

D. 3 to 5 times per week

E. Approximately once per day

Physical Self-Esteem Scale

Serial number	Item	①Completely Disagree ②Mostly Disagree ③Somewhat Agree ④Completely Agree			
1	I feel extremely proud of my body shape and physical abilities.	1	2	3	4
2	I am sometimes dissatisfied with my physical condition or fitness.	1	2	3	4
3	I do not feel very confident about my body.	1	2	3	4
4	I am very satisfied with my physical abilities.	1	2	3	4
5	I wish I could care more about my body.	1	2	3	4
6	I feel very satisfied with my body shape.	1	2	3	4
7	I feel I am not capable of handling most sports activities.	1	2	3	4

8	I rank among the best when it comes to physical abilities.	1	2	3	4
9	I do not feel very confident when participating in sports.	1	2	3	4
10	I am always one of the best when participating in sports.	1	2	3	4
11	I sometimes learn new movements more slowly than others.	1	2	3	4
12	I am always the first to join physical activities whenever I have the chance.	1	2	3	4
13	I do not feel confident about my physical condition and fitness level.	1	2	3	4
14	I can regularly engage in energetic physical activities.	1	2	3	4
15	Compared to most people, I often struggle to maintain good energy and fitness levels.	1	2	3	4
16	I feel a bit uncomfortable in fitness or exercise environments.	1	2	3	4
17	I feel very confident about my physical condition and abilities.	1	2	3	4
18	Compared to most people, I always maintain a high level of physical fitness.	1	2	3	4

19	Compared to most people, I have a highly attractive body.	1	2	3	4
20	I find it difficult to maintain an attractive body.	1	2	3	4
21	I feel uncomfortable when wearing very little clothing.	1	2	3	4
22	Others always envy me because of my outstanding physique or figure.	1	2	3	4
23	Compared to most people, my body shape does not look the best.	1	2	3	4
24	I feel very confident about my physical appearance.	1	2	3	4
25	My body is much stronger than that of most people of the same gender.	1	2	3	4
26	Compared to most people of the same gender, I run faster.	1	2	3	4
27	I feel very confident when it comes to speed in sports.	1	2	3	4
28	I feel a lack of confidence when it comes to physical strength.	1	2	3	4
29	Compared to most people of the same gender, I have much better explosive power.	1	2	3	4
30	In situations that require physical strength, I feel I	1	2	3	4

	am not as good as others.				

Exercise Motivation Scale

Serial number	Item	①Completely Disagree<br>②Disagree<br>③Somewhat Disagree<br>④Neutral<br>⑤Somewhat Agree<br>⑥Agree<br>⑦Completely Agree						
1	Because I feel happy during exciting experiences.	1	2	3	4	5	6	7
2	Because sports activities allow me to learn more about the sport I participate in, which brings me a lot of joy.	1	2	3	4	5	6	7
3	I have always had good reasons to exercise, but now I am not sure whether I should continue.	1	2	3	4	5	6	7
4	To experience the joy of discovering new exercise methods.	1	2	3	4	5	6	7
5	I don't know; it seems I cannot achieve success in the sports activities I participate in.	1	2	3	4	5	6	7
6	Because sports activities earn me respect from people I know.	1	2	3	4	5	6	7
7	I think sports activities are one of the best ways to connect with others.	1	2	3	4	5	6	7
8	Because mastering difficult sports techniques gives me a sense of self-satisfaction.	1	2	3	4	5	6	7
9	I believe participating in sports activities is necessary to maintain good health.	1	2	3	4	5	6	7
10	For the reputation of being an athlete.	1	2	3	4	5	6	7
11	Because sports activities are the best way I choose to promote my development in other areas.	1	2	3	4	5	6	7
12	To experience the joy of overcoming some of my weaknesses.	1	2	3	4	5	6	7

13	To feel the excitement of being fully engaged in sports activities.	1	2	3	4	5	6	7
14	Because I must engage in sports activities to achieve a good sense of self.	1	2	3	4	5	6	7
15	To feel satisfaction when I improve my abilities.	1	2	3	4	5	6	7
16	Because the people around me believe a healthy body is very important.	1	2	3	4	5	6	7
17	Through sports activities, I can learn many things that are beneficial for other aspects of life.	1	2	3	4	5	6	7
18	Because when I engage in my favorite sports activities, I feel a strong sense of passion.	1	2	3	4	5	6	7
19	I am not sure now, but I do think my body and sports activities are unrelated.	1	2	3	4	5	6	7
20	Because I feel joy when I complete challenging movements.	1	2	3	4	5	6	7
21	If I don't spend some time on sports activities, I feel uneasy.	1	2	3	4	5	6	7
22	To show everyone how good I am at sports activities.	1	2	3	4	5	6	7
23	To enjoy learning new techniques I haven’t practiced before.	1	2	3	4	5	6	7
24	Because sports activities are the best way for me to maintain good relationships with friends.	1	2	3	4	5	6	7
25	Because I enjoy the feeling of being fully immersed in an activity.	1	2	3	4	5	6	7
26	Because I must exercise regularly.	1	2	3	4	5	6	7
27	To feel the joy of discovering new activity strategies.	1	2	3	4	5	6	7
28	I often tell myself: It seems I cannot achieve the goals I set for myself.	1	2	3	4	5	6	7

Social support scale

**Serial number**	**Item**	**①Completely Disagree**<br>**②Disagree**

		③Somewhat Agree<br>④Agree<br>⑤Completely Agree				
1	My family can provide me with tangible and practical help.	1	2	3	4	5
2	When I need it, I can receive emotional help and support from my family.	1	2	3	4	5
3	I can discuss my problems with my family.	1	2	3	4	5
4	My family is willing to help me make various decisions.	1	2	3	4	5
5	My friends can genuinely help me.	1	2	3	4	5
6	In times of difficulty, I can rely on my friends.	1	2	3	4	5
7	My friends can share both happiness and sorrow with me.	1	2	3	4	5
8	I can discuss my problems with my friends.	1	2	3	4	5
9	When I encounter problems, some people (teachers, classmates) will be there for me.	1	2	3	4	5
10	I can share both happiness and sorrow with certain people (teachers, classmates).	1	2	3	4	5
11	When I am in trouble, some people (teachers, classmates) are a true source of	1	2	3	4	5

	comfort for me.					
12	Certain people (teachers, classmates) in my life care about my feelings.	1	2	3	4	5

The questionnaire is now complete. Thank you for your responses!
