# Supplementary material for: From physical self-esteem to sports participation: The mediating role of exercise motivation and social support in adolescents
Source: PLoS One. 2025 May 22;20(5):e0324588. doi: 10.1371/journal.pone.0324588 (PMC12097568; doi:10.1371/journal.pone.0324588)
Supplement: Attachment — Submitted filename: Response to Reviewers.docx [file pone.0324588.s003.docx]

Response to Reviewers

We would like to express our sincere gratitude to both reviewers for their thorough and constructive comments. Your insightful feedback has been invaluable in helping us improve the quality, rigor, and clarity of our manuscript. We carefully reviewed all the comments and have made substantial revisions to address the concerns raised. Below, we provide a point-by-point response to each of the reviewers’ comments, detailing the revisions we have made.

Once again, we are deeply grateful for your time, expertise, and constructive suggestions, which have significantly strengthened our study. We hope that the revised manuscript meets your expectations.

**Reviewer 1**

**Comment 1:**Please check tables 2, 4, and 8.
**Reviewer’s Comme**nt**:**Tables 2,4, and 8 - please double check and remove "squares" and replace with the correct one in the tables indicated.

**Respo**nse**:**Thank you for your valuable suggestions and thorough evaluation of our research. Your feedback has helped us critically reflect on the issues in the study and provided clear directions for further improvements. In response to your suggestion, we have carefully reviewed Tables 2, 4, and 8 and have replaced the incorrect use of "squares" with the correct terminology to ensure accuracy and adherence to academic standards.

**Revisions Made:**

**Table 2.** Scales used in this study.

| **Scale** | **Author (Year)** | **Item quantity** | **Scoring** | **Dimensions** |
| --- | --- | --- | --- | --- |
| Physical self-esteem | **Xia Xu(2001)** | 30 | 4 | **Physical self-worth;**  **Sport ability;**  **Physical condition;**  **Physical attractiveness;**  **Physical fitness** |
| Physical exercise | Deqing Liang (1994) | 3 | 5 | Exercise intensit; Duratio;  exercise Frequency |
| Exercise motivation | Liwei Zhang  （2004） | 28 | 7 | **Intrinsic motivation;**  **Extrinsic motivation;**  **Amotivation** |
| Social support | Wei Chen(2016) | 12 | 5 | **Family;**  **Friends;**  **Others** |

**Table 4.** Comparative fit indices for alternative factor structures of key constructs.

| **Model** | **Factor** | **χ 2** | ***df*** | **Delta χ2**  **(Delta *df*)** | **CFI** | **TLI** | **SRMR** | **RMSEA (90%CI)** |
| --- | --- | --- | --- | --- | --- | --- | --- | --- |
| Four-factor model | PSE,EM,SS,SP | 363.16 | 71 | - | 0.980 | 0.974 | 0.025 | 0.040 (0.036-0.044) |
| Three-factor model | PSE+EM,SS,SP | 2002.29 | 74 | -0.113  (-0.137) | 0.867 | 0.837 | 0.078 | 0.100 (0.097-0.104) |
| Two-factor model | PSE+EM+SS,SP | 3186.75 | 76 | -0.194  (-0.231) | 0.786 | 0.743 | 0.089 | 0.126 (0.122-0.130) |
| One-factor model | PSE+EM+SS+SP | 3722.29 | 77 | -0.231  (-0.271) | 0.749 | 0.703 | 0.093 | 0.135 (0.132-0.139) |
| PSE,Physical Self-Esteem. SP, Sports participation. EM, Exercise Motivation. SS,Social Support.All △χ 2 passed the significance test at 0.01 level. | | | | | | | | |

**Table 8.** Testing for structural invariance across gender.

|  | **χ 2/*df*** | **CFI** | **Delta CFI** | **TLI** | **Delta**  **TLI** | **SRMR** | **RMSEA**  **(90%CI)** |
| --- | --- | --- | --- | --- | --- | --- | --- |
| Unconstrained | 3.079 | 0.982 | - | 0.976 | - | 0.029 | 0.028 (0.025-0.032) |
| Measurement weights | 3.128 | 0.980 | -0.002 | 0.975 | -0.001 | 0.030 | 0.029 (0.025-0.032) |
| Structural weights | 3.092 | 0.980 | -0.002 | 0.976 | 0.000 | 0.032 | 0.028 (0.025-0.032) |
| Structural covariances | 3.070 | 0.980 | -0.002 | 0.976 | 0.000 | 0.032 | 0.028 (0.025-0.032) |
| Structural residuals | 3.023 | 0.980 | -0.002 | 0.977 | +0.001 | 0.032 | 0.028 (0.025-0.031) |

**Specific Modifications:**

We carefully reviewed Tables 2, 4, and 8, and corrected the incorrect symbols in the tables to ensure that all terms are accurate and consistent, in accordance with academic standards.

**Comment 2:**Check punctuation marks.
**Reviewer’s Comme**nt**:**Please also double check the text for punctuation mistakes.

**Respo**nse**:**Thank you for your valuable suggestions and in-depth evaluation of our research. Your feedback has helped us critically reflect on the issues in our study and provided clear directions for further improvement. In response to your suggestion, we have carefully checked the punctuation throughout the manuscript and made corrections to any inappropriate punctuation to ensure accuracy and compliance with academic standards.

**Revisions Made:**

**1 Introduction**

**Specific Modifications:**

Removal of the Colon (":") after "1 Introduction":The original text included the colon after the title "1 Introduction:".The colon has been removed to adhere to academic standards and formatting guidelines for section headings. In most academic writing styles, section titles do not require a colon unless they are followed by a subtitle. Since there is no subtitle following "Introduction," the colon is unnecessary.This adjustment improves the presentation of the section heading and aligns it with proper academic formatting conventions.

**Comment 3:**Limitations and future directions.
**Reviewer’s Comme**nt**:**In point 6 Limitations and future directions, please elaborate what kind of the objective measures of sports participation the Authors have on their mind, to give a clear directions for future research, and demonstrate a solid understanding of the focused field.

**Respo**nse**:**Thank you for your valuable suggestions and in-depth evaluation of our study. Your feedback has prompted us to critically reflect on the issues in our research and provided clear directions for further improvement. In response to your suggestions, we have elaborated in Section 6 "Limitations and Future Directions" on the objective measures that can be adopted in future research to better assess sports participation. We have explicitly proposed the use of activity trackers, wearable devices (such as smartwatches), and behavioral observations to collect more accurate data, thereby enhancing the reliability of the study.

**Revisions Made:**

## **6 Limitations and future directions**

Although this study provides valuable insights, there are some limitations that should be acknowledged. First, the cross-sectional design of this study limits the ability to infer causal relationships. Future longitudinal studies could more deeply explore the dynamic relationships between these variables over time, allowing for a more accurate understanding of causal pathways and long-term effects.

Second, this study relied on self-reported measures, which may introduce biases such as social desirability bias or recall bias. To reduce these biases, future research could incorporate objective measures of sports participation, such as activity trackers, wearable devices (e.g., smartwatches), or behavioral observations to obtain more precise data. These tools could provide multidimensional information on the frequency, duration, and intensity of adolescent physical activity, enhancing the accuracy and reliability of the data.

Additionally, future research could further explore other potential mediators, particularly psychological health factors (e.g., anxiety, depression symptoms) and environmental factors (e.g., family support, school resources). By incorporating these factors, researchers can gain a more comprehensive understanding of how physical exercise influences adolescents' psychological resilience and self-efficacy through multiple mechanisms, providing theoretical support for more effective intervention strategies.

**Specific Modifications:**

1:Clearer objective measurement tools: We have mentioned that future research should use objective measurement tools such as activity trackers, wearable devices (e.g., smartwatches), and behavioral observations. These tools can more accurately record and analyze various parameters of physical activity.

2:A deeper exploration of future research directions: We have expanded the discussion on potential mediators to be included in future research, particularly the roles of psychological health and environmental factors. This will help provide a more comprehensive understanding of the relationship between physical exercise and psychological development.

3:Strengthened recommendations for causal inference: By proposing longitudinal studies, we have clearly articulated the need to explore the dynamic changes in causal relationships, enhancing the depth and feasibility of future research.

We sincerely appreciate your insightful comments, which have greatly enhanced the clarity, accuracy, and overall presentation of the manuscript. Thank you for your valuable feedback.

**Reviewer 2**

**Comment 1:**Advantages of the manuscript.
**Reviewer’s Comme**nt**:**The manuscript presents an interesting study examining the links between physical self-esteem, exercise motivation, social support, and sports participation. The authors use advanced structural equation modelling to explore the connections and also test for the moderating role of gender. The advantages of the study include the large sample size, the innovative conceptual framework, ethical execution of the study, and the advanced analytic techniques.

**Response:**We sincerely appreciate the reviewer’s thoughtful and constructive feedback. Thank you for recognizing the strengths of our study, including the large sample size, the innovative conceptual framework, the ethical execution of the study, and the advanced analytic techniques employed. Your positive comments on the use of structural equation modeling and the exploration of the moderating role of gender are greatly valued. We are grateful for your detailed review, which has not only highlighted the merits of our work but also provided us with important perspectives for enhancing the quality and depth of our research. Your input has been invaluable in guiding the further refinement of our manuscript.

**Comment 2:**The argument about the importance of sports participation, as the key outcome, should be beefed up.
**Reviewer’s Comme**nt**:**The argument about the importance of sports participation, as the key outcome, should be beefed up. Now, the authors spend more time on the interrelations between the four factors of interest but do not stress enough the importance of sports participation for various adolescent outcomes.

**Respo**nse**:**Thank you for your valuable comments and in-depth evaluation of our study. Your feedback has prompted us to reflect more deeply on the focus of our research and has enhanced our understanding of the importance of sports participation. In response to your suggestions, we have made comprehensive revisions to the relevant content, further strengthening the discussion of sports participation as a key outcome, and emphasizing its critical role in adolescent mental health, emotional regulation, and social skills. We believe that, with these adjustments, the discussion section now more effectively balances the interrelationships among the four factors and highlights the profound impact of sports participation on various aspects of adolescent development.

**Revisions Made:**

# **5** Discussion

This study provides valuable insights into the mechanisms linking physical self-esteem to sports participation among adolescents. The results validate the hypothesized relationships and emphasize the critical roles of exercise motivation and social support in fostering sports engagement. While we discuss the interrelations among the four key factors, this study’s major contribution lies in uncovering the multidimensional impact of sports participation on adolescents, particularly in enhancing psychological, social, and academic outcomes. Below, we delve into the key findings, comparing them to existing literature and exploring their practical implications.

## **5.1 Physical self-esteem as a predictor of sports participation**

The results confirm that physical self-esteem significantly and positively predicts adolescents’ sports participation (H1). This finding aligns with previous research highlighting the importance of self-perception in motivating sports engagement [6, 25]. Adolescents with higher physical self-esteem tend to feel more confident in their physical abilities, which subsequently encourages greater participation in sports. This underscores the importance of interventions aimed at enhancing physical self-esteem, such as skill-building programs and positive reinforcement in sports settings. More importantly, sports participation, as a key outcome, has been shown to improve not only physical fitness but also psychological well-being and social competence among adolescents.

## **5.2 Mediating role of exercise motivation**

The mediating role of exercise motivation between physical self-esteem and sports participation (H2) indicates that higher physical self-esteem enhances adolescents' intrinsic or extrinsic motivation to exercise, thereby increasing their level of sports participation. Exercise motivation explained 33.26% of the total effect, underscoring its importance as a mediating pathway. More deeply, sports participation, as a key outcome, is closely related to improvements in psychological health, emotional regulation, and social skills. According to self-determination theory [4], motivation is the core factor driving behavior. Encouraging adolescents to engage in sports not only enhances physical health but also promotes the development of emotional and social skills. Future interventions should focus on cultivating adolescents' intrinsic motivation, particularly by enhancing their motivation to participate through the enjoyment of physical activities, which will further promote their overall development.

## **5.3 Mediating role of social support**

Social support also plays a significant mediating role between physical self-esteem and sports participation (H3), explaining 16.74% of the total effect. Sports participation is not only a result of individual factors but is also influenced by the social environment, including family, friends, and peers [16,33,49]. Social support indirectly promotes adolescents' physical self-esteem and exercise motivation by increasing opportunities and confidence to participate in physical activities. Research has shown that social support not only enhances motivation for sports participation but also improves adolescents' emotional health and promotes psychological resilience. To amplify this effect, family-oriented sports activities, peer mentoring, and the establishment of social support networks are particularly important. These measures help increase adolescents' motivation to engage in physical activities, thereby having a profound impact on their psychological development.

## **5.4 Sequential mediation of exercise motivation and social support**

The sequential mediation pathway through both exercise motivation and social support (H4) further underscores the interconnected nature of these mediators. This pathway accounted for 29.61% of the total effect, indicating that physical self-esteem influences sports participation not only through individual motivation but also through the social context. These findings highlight the complexity of adolescent sports behavior and emphasize the need for comprehensive strategies that address both personal and social factors. Sports participation in this context is seen not only as a way to improve physical health but also as an essential factor in enhancing social skills, emotional resilience, and academic achievement.

## **5.5 Demographic differences**

This study reveals that gender, grade level, and family location significantly affect adolescents' physical self-esteem, exercise motivation, social support, and sports participation levels. Males tend to score higher than females on all variables, potentially due to societal expectations and cultural reinforcement of male sports participation. High school students exhibit stronger physical self-esteem and exercise motivation, while middle school students tend to rely more on social support. Rural adolescents demonstrate higher participation rates and stronger support networks, likely due to better access to sports resources and stronger community interaction in rural areas. Existing studies often focus on single-variable analyses, neglecting the dynamic relationships between gender, grade level, and urban-rural disparities [14, 27]. By integrating a multidimensional model, this study systematically uncovers the comprehensive impacts of demographic variables on sports participation, offering theoretical support for interventions tailored to specific group needs. For instance, interventions targeting female adolescents can focus on enhancing physical self-esteem and intrinsic motivation, while urban adolescents may benefit from community-based activities to strengthen social support.

## **5.6 Implications for practice and policy**

The findings of this study provide not only guidance for practical interventions but also valuable insights for policymakers in promoting adolescent sports participation. Firstly, the central role of physical self-esteem in adolescent sports participation highlights the need for a stronger focus on enhancing adolescents' physical self-esteem in practice. Schools and community programs should work towards developing adolescents' confidence in their physical abilities through skill-building, positive feedback, and body-positive campaigns. Targeted interventions for adolescents with lower self-esteem can effectively boost their confidence, thereby motivating them to participate in sports activities.

Secondly, fostering exercise motivation is crucial in promoting adolescent sports participation. Policymakers should prioritize the cultivation of intrinsic motivation, particularly by emphasizing the enjoyment and personal achievement that comes from sports. For example, offering a variety of sports programs can encourage adolescents to engage in physical activities while enhancing their long-term interest and motivation. Moreover, extrinsic motivation, such as rewards and recognition, can also play a role in encouraging sports participation, particularly for short-term engagement.

In terms of social support, this study underscores the significant role that family, peers, and community networks play in promoting adolescent sports participation. Therefore, policies should encourage collaboration among families, schools, and communities to create a supportive environment for adolescents. Governments and educational authorities should actively promote initiatives such as family sports days and peer support groups to provide adolescents with the social support they need to overcome barriers to sports participation. Particularly in regions with significant urban-rural disparities, policies should ensure that all adolescents have access to adequate social support and resources, which would help achieve higher participation rates.

Lastly, this study reveals that adolescent sports participation is significantly influenced by factors such as gender, grade level, and geographic location. In response, policies should adopt more inclusive and targeted measures, especially gender-sensitive strategies, to reduce participation gaps among different groups. For example, interventions aimed at female adolescents can focus on enhancing physical self-esteem and fostering intrinsic motivation, while urban adolescents may benefit more from community-based activities that strengthen their social support networks.

In conclusion, policymakers should consider the multifaceted factors influencing adolescent sports participation, including individual, family, and social support. Policies should not only focus on providing sports resources and opportunities but also promote the psychological and emotional development of adolescents to ensure comprehensive support for their participation in sports activities. Through these multi-level policy interventions, we can create a more inclusive and supportive environment for adolescent sports participation, fostering their overall physical and mental development.

**Specific Modifications:**

1: We emphasized the importance of sports participation: In the revised version, we not only analyzed the interrelationships among physical self-esteem, exercise motivation, social support, and sports participation, but also discussed in depth the profound impact of sports participation on adolescent mental health, social adaptation, academic performance, and other aspects.

2: We strengthened the combined role of individual and social factors: By exploring the mediating roles of exercise motivation and social support, we further revealed how sports participation promotes adolescent development through both individual and social factors.

3: We provided more targeted intervention strategies: In discussing gender differences and demographic factors, we provided theoretical support for designing customized interventions for different groups, emphasizing the necessity of personalized sports promotion strategies.

**Comment 3:**The wording in the introduction is inappropriate.
**Reviewer’s Comme**nt**:**The introduction (page 9) states “research on how these three factors (physical self-esteem, exercise motivation, and social support) interact and jointly influence…”. The term “interact” is inappropriate here since it refers to statistical interaction which is not the analytic focus here.

**Respo**nse**:**Thank you for your valuable feedback and thorough evaluation of our manuscript. We greatly appreciate your suggestion regarding the term "interact" in the introduction. We now understand that the term might imply a statistical interaction, which was not the focus of our analysis. In response to your comment, we have revised the phrasing to better reflect the nature of the relationships among the factors. The revised sentence now accurately describes how physical self-esteem, exercise motivation, and social support jointly influence sports participation, without implying statistical interaction. Thank you again for helping us improve the clarity and precision of our manuscript.

**Revisions Made:**

In sports activities, exercise motivation and social support are two important psychological and social factors, both playing a significant role in adolescents' sports participation [11,12]. Exercise motivation determines an individual's intrinsic drive and persistence in engaging in sports activities [13], and research has shown that adolescents with higher intrinsic motivation are more likely to continue participating in sports and experience greater psychological and physical benefits [14,15]. Meanwhile, social support—especially emotional support from family, peers, and coaches—is considered to provide essential encouragement and motivation, enhancing adolescents' confidence and willingness to engage in sports activities [16-18]. Although existing research has gradually revealed the role of exercise motivation and social support in sports participation [19,20], research on how these three factors (physical self-esteem, exercise motivation, and social support) jointly influence sports participation remains insufficient.

**Specific Modifications:**

1:Replacement of the term "interacting": We have modified the term "interact" in the introduction to avoid misleading readers by confusing it with statistical interaction. Specifically, the original phrase "how these three factors (physical self-esteem, exercise motivation, and social support) interact" has been revised to "how these three factors jointly influence sports participation." This change ensures the accuracy of the terminology and avoids the inappropriate use of statistical analysis terms.

2:Revised expression: In the revised text, we have clearly stated "how these three factors (physical self-esteem, exercise motivation, and social support) jointly influence sports participation," and further emphasized the importance and interaction of these factors in adolescents' sports participation, thereby more clearly conveying the focus of the study.

**Comment 4:**The authors introduce physical self-esteem without introducing the definition of this factor.
**Reviewer’s Comme**nt**:**The authors introduce physical self-esteem without introducing the definition of this factor. Also, I am wondering whether there is a need to introduce self-esteem as a broader construct before specialising further. Similarly, exercise motivation is presented as a broad concept. Yet, motivation is not a unitary concept; there are several theories of motivation (e.g., goals, intrinsic motivation vs. extrinsic, value). Latter on, the authors present the concept of intrinsic motivation in that particular section. I would advise to refine and define the concept before delving deeper into the relevant literature.

**Respo**nse**:**Thank you for your valuable suggestions and thorough evaluation of our research. Your feedback prompted us to revisit the definitions of physical self-esteem and exercise motivation, and we have further refined the explanation of these concepts in the manuscript. In response to your suggestions, we have provided a clear definition of "physical self-esteem" in Section 2.1 to ensure that readers can easily understand this concept. Additionally, in Section 2.2.1, where we introduce exercise motivation, we have provided a more precise definition, clearly distinguishing between intrinsic and extrinsic motivation and briefly outlining the relevant theoretical background. Through these revisions, we aim to present these key concepts more clearly and lay a stronger foundation for the subsequent literature review and analysis. Once again, we greatly appreciate your valuable feedback, which has significantly enhanced the rigor and clarity of our study.

**Revisions Made:**

**2.1 The relationship between adolescent physical self-esteem and sports participation**

Physical self-esteem is a complex and multidimensional concept that encompasses various components, including perception, cognition, emotion, and behavior[22]. Its perceptual dimension is typically referred to as body self-esteem, which is defined as an individual’s subjective evaluation of their body[23]. Originally, body self-esteem was defined as the evaluation of various aspects of one’s body within the framework of social judgment; as a part of overall self-esteem, it includes both a general sense of self-worth regarding the body and more specific satisfaction with various bodily aspects, involving individual cognitive, emotional, and evaluative processes[24]. Some researchers have further defined body self-esteem as “the satisfaction or dissatisfaction with various aspects of one’s physical appearance,” emphasizing its close relationship with social evaluation, and considering it an important component of self-esteem[25]. Additionally, other scholars have viewed body self-esteem more positively, associating it with a positive body image, which involves the emotions evoked by an individual’s subjective perception and evaluation of their body, and emphasizing the importance of appreciating and caring for one's body[26].

During adolescence, body self-esteem, as an important component of self-concept, influences psychological state and behavioral performance[27]. Higher body self-esteem is often associated with more positive behavioral choices in adolescents, particularly in terms of participation in physical activities[28,29]. Specifically, body self-esteem enhances adolescents' initiative and persistence in sports by boosting their self-confidence and self-acceptance[30,31]. Additionally, body self-esteem helps reduce anxiety and self-rejection caused by dissatisfaction with body image, thereby increasing adolescents' interest in and psychological readiness for sports participation[32-34]. Furthermore, body self-esteem plays a crucial role in emotional regulation and the enhancement of self-efficacy, with these factors collectively influencing adolescents' attitudes and behaviors toward physical activities[35-37]. Through these psychological mechanisms, body self-esteem provides positive psychological resources that can impact adolescents' performance and sustained engagement in sports[38].

**2.2.1 Exercise motivation**

Motivation is the intrinsic drive that prompts individuals to engage in a particular activity. It stimulates the willingness to participate and reinforces the individual’s behavioral motivation on a psychological level[39]. In psychology, the initiation and maintenance of behavior are driven by continuous motivation. Motivation not only reflects the effort an individual exerts to achieve goals but also represents the primary drive formed to meet spiritual, physical, or social needs[40]. The formation of motivated behavior is closely linked to autonomy needs, with motivation existing at three levels: amotivation, extrinsic motivation, and intrinsic motivation. Extrinsic motivation includes internalization, identification, and external regulation, while intrinsic motivation encompasses curiosity, achievement, and stimulation. Research shows that the stronger the intrinsic motivation, the greater the individual’s interest, motor skills, and learning outcomes in sports. Therefore, the different levels of motivation have significant effects on sports outcomes and psychological states[40].In this study, exercise motivation is defined as the intrinsic drive and external factors that prompt individuals to participate in sports activities, encompassing their interest, desire, and goals related to sports. Exercise motivation encourages individuals to actively engage in physical exercise and participate in various sports events.

Physical self-esteem indirectly influences adolescents' exercise motivation by enhancing their confidence and self-acceptance [30,31]. Adolescents with higher levels of self-esteem tend to view sports activities as a way to enhance their self-identity and self-efficacy, which provides psychological support and stimulates stronger exercise motivation [41,42]. In addition, physical self-esteem helps regulate emotions and reduces negative feelings arising from dissatisfaction with body image, improving emotional regulation abilities and creating favorable conditions for enhancing exercise motivation [37,43]. Physical self-esteem is also closely related to self-efficacy, and higher levels of self-esteem boost adolescents' confidence in sports activities, further promoting their exercise motivation and sustained participation [44-46] The enhancement of exercise motivation plays an important role in sports participation. Studies have shown that stronger intrinsic motivation significantly increases the frequency and quality of sports participation [47-48]. Therefore, physical self-esteem, by boosting exercise motivation, provides essential psychological support for adolescents' sports participation, which may, in turn, impact the sustainability and effectiveness of their long-term engagement.

**Specific Modifications:**

1:Clarification of the definition of "physical self-esteem": Based on your suggestion, we have provided a clear definition of the concept of "physical self-esteem" in Section 2.1 to ensure that readers can accurately understand this factor. We clarified the multidimensional structure of physical self-esteem, including its perceptual, cognitive, emotional, and behavioral components, to help readers better understand its importance in this study.

2:Precise definition of the concept of "exercise motivation": In response to your comment regarding the broad nature of "exercise motivation," we have provided a more precise definition of this concept. Specifically, we clearly differentiate between intrinsic and extrinsic motivation and introduced relevant theoretical backgrounds, including self-determination theory, the differences between intrinsic and extrinsic motivation, and the diversity of motivation. This modification ensures a full understanding of the concept of motivation and avoids a simplified interpretation.

**Comment 5:**I find it difficult to locate all the hypotheses interspersed inside the literature review.
**Reviewer’s Comme**nt**:**I find it difficult to locate all the hypotheses interspersed inside the literature review. Please consider transferring all hypotheses to a ‘present study’/ ‘theoretical model’ section.

**Respo**nse**:**Thank you for your valuable suggestions and thorough evaluation of our study. Your feedback prompted us to re-examine the structure of the paper and further optimize the presentation of the hypotheses. Following your suggestion, we have moved all hypotheses from the literature review section to the "Theoretical Model" section. This change not only clarifies the structure of the paper but also helps to better present the theoretical framework and hypotheses of the study. We believe this modification enhances the logical flow and readability of the paper. Once again, we greatly appreciate your constructive comments, which have significantly improved the organization and clarity of this study.

**Revisions Made:**

**2.4 Theoretical model**

Based on the existing literature on the relationships between physical self-esteem, exercise motivation, social support, and adolescent sports participation, this study integrates relevant theoretical and empirical research findings to propose a comprehensive theoretical framework (see Figure 1), aimed at exploring the following aspects:

**1.Direct relationship:** The direct impact of adolescent physical self-esteem on sports participation.

**2.Mediating effect:** The mediating role of exercise motivation and social support in the relationship between physical self-esteem and sports participation.

**3.Chain mediation effect:** The interaction between exercise motivation and social support, highlighting how these two factors jointly influence the sustainability and extent of adolescents' sports participation.

Figure 1. Theoretical model of the impact of adolescent physical self-esteem on sports participation: the chain mediation effect of exercise motivation and social support.

This model hypothesizes that adolescent physical self-esteem influences sports participation directly or indirectly through factors such as exercise motivation and social support. Exercise motivation and social support may play important mediating roles in this process, facilitating the impact of physical self-esteem on sports participation. By integrating these factors, this framework provides a systematic theoretical perspective on the multidimensional influences on adolescents' sports participation, and offers theoretical support and practical guidance for developing targeted interventions.

Based on the above, the hypotheses of this study are as follows:

**Hypothesis1(H1):**Physical self-esteem significantly positively predicts adolescents' sports participation.

**Hypothesis2(H2):**Exercise motivation mediates the relationship between physical self-esteem and sports participation in adolescents.

**Hypothesis3(H3):**Social support mediates the relationship between physical self-esteem and sports participation in adolescents.

**Hypothesis4(H4):**Exercise motivation and social support mediate the relationship between physical self-esteem and sports participation in adolescents.

**Specific Modifications:**

Reorganization and repositioning of the hypotheses: Following your suggestion, we have moved all hypotheses from the literature review section to the "Theoretical Model" section. This adjustment centralizes the presentation of the hypotheses, making it easier for readers to clearly understand the theoretical framework of the study and its associated hypotheses.

**Comment 6:**Regarding power analysis
**Reviewer’s Comme**nt**:**Regarding power analysis, I am afraid that I fail to see the relevance of power analysis here considering that power analysis was done only for multiple regression, whereas the authors used SEM modelling. SEM modelling power analysis requires simulation.

**Respo**nse**:**We appreciate the reviewer’s insightful comment. In response, we have revised the power analysis section of the manuscript. Initially, the power analysis was performed using GPower, which is commonly applied in regression-based analyses. However, as per the reviewer’s suggestion, we have now conducted the power analysis using semPower, a tool specifically designed for SEM power analysis that incorporates simulation-based methods. This allows for a more appropriate assessment of power in the context of structural equation modeling.

For clarity, we report the sample size estimation based on the semPower analysis, which indicated that a minimum of 243 participants would be needed to achieve adequate power (desired power of 0.80) for detecting significant effects in the SEM model. This new analysis replaces the previous use of GPower and aligns with best practices for SEM research. We have also maintained the rule of thumb based on the number of questionnaire items, which provides additional sample size guidance.

For reference, the power analysis was conducted using the parameters outlined in Moshagen and Bader (in press) for semPower (Moshagen, M., & Bader, M. (in press). semPower: General Power Analysis for Structural Equation Models. Behavior Research Methods. [https://doi.org/10.3758/s13428-023-02254-7](https://doi.org/10.3758/s13428-023-02254-7)). The tool's capabilities for simulation-based power analysis offer a robust and fitting approach for SEM studies.

We have updated the manuscript to reflect this change and have removed the previous GPower analysis, ensuring that the power analysis is now consistent with the structural equation modeling approach used in the study.

**Revisions Made:**

**3.1.1 Sample size justification**

To ensure the robustness of the research design and the reliability of the results, this study estimated the required sample size using semPower for structural equation modeling (SEM) and the rule of thumb commonly applied in social science research.

First, a power analysis for SEM was conducted using semPower[66], with the following parameters: desired power of 0.80, alpha level of 0.05, RMSEA as the effect measure, an effect size of 0.05, degrees of freedom (df) of 50, and 12 manifest variables. Under these conditions, the analysis indicated that at least 243 participants would be needed to detect a significant effect in the structural equation model. semPower analysis ensures that, with the preset statistical power, the relationships among physical self-esteem, exercise motivation, social support, and sports participation can be accurately assessed.

Second, according to the rule of thumb in social science research, it is recommended that the sample size be 10 to 15 times the total number of items in the questionnaire. The measurement tools used in this study included: the physical self-esteem scale (30 items), the sports participation scale (3 items), the exercise motivation scale (28 items), and the social support scale (12 items). Therefore, the estimated sample size range was between 730 and 1,095 participants (i.e., 73 items × 10 to 73 items × 15). This method further ensures that the sample size is scientifically appropriate and comprehensive in the actual survey design.

Based on these two methods, the minimum required sample size for this study was 243 participants based on SEM power analysis, while the ideal sample range based on the questionnaire rule of thumb was 730 to 1,095 participants. During the actual data collection process, to enhance the statistical power and external validity of the research results, data from 2,588 adolescent participants were ultimately collected. This large sample size significantly exceeds the minimum requirements set by both the power analysis and the rule of thumb, providing sufficient data support for analyzing the relationships among the research variables. Furthermore, exceeding the minimum sample size requirement improves the stability, generalizability, and representativeness of the findings across different demographic characteristics.

**Specific Modifications:**

1:**Change of Power Analysis Tool.** We originally used the GPower tool for power analysis, which is commonly applied in regression-based analyses. Based on your suggestion, we have now switched to using semPower, a tool specifically designed for SEM power analysis that incorporates simulation-based methods. With this change, we have adopted a more appropriate power analysis method for SEM research, ensuring that the power evaluation is better suited to our study's model.

2:**Revised Description of Power Analysis.** In the manuscript, we have clearly reported the sample size estimation based on the semPower analysis, which indicated that at least 243 participants would be needed to detect significant effects in the SEM model with a desired power of 0.80. We also explained that this new power analysis method replaces the previous use of GPower and aligns with best practices for SEM research.

3:**Retention of the Rule of Thumb.** In addition to the semPower analysis, we have retained the rule of thumb based on the number of questionnaire items, which provides additional guidance for sample size. We kept this method to enhance the scientific rigor and comprehensiveness of the sample size estimation.

4:**Provision of References and Tool Explanation.** In the revised manuscript, we referenced the work of Moshagen and Bader and explicitly stated the parameters used in the semPower analysis. This citation provides the theoretical basis for the power analysis tool and further demonstrates its suitability for SEM research.

**Comment 7:**Multistage cluster sampling.
**Reviewer’s Comme**nt**:**There is a mention of multi-stage cluster sampling, yet it is unclear whether this kind of sampling design influences the variables under study. Perhaps, it makes sense here to report the intra-class correlation coefficient per key variable to understand the potential impact of ignoring the cluster sampling.

**Respo**nse**:**We would like to express our gratitude to the reviewer for their insightful comment. In response, we have provided additional details regarding the multi-stage cluster sampling design and its potential impact on the study's key variables. Specifically, we calculated intra-class correlation coefficients (ICC) for each key variable to assess the clustering effects. The results indicated minimal clustering influence, with school-level ICC values ranging from 0.5% to 1.1%, and classroom-level ICC values ranging from 0.9% to 1.8%.

Given these results, we adjusted our statistical analyses using Huber-White robust standard errors, a widely accepted method for correcting clustering effects. This adjustment ensures that the relationships among the variables—such as physical self-esteem, exercise motivation, social support, and sports participation—are accurately estimated, with proper consideration of the hierarchical structure in the data.

These revisions strengthen the manuscript by clearly outlining the sampling design and the steps taken to account for potential clustering effects, thereby ensuring the validity and reliability of the study’s findings.

**Revisions Made:**

**3.1.2 Participant selection process**

The participants in this study were students from grades 7 to 12, including both junior high school (grades 7 to 9) and senior high school (grades 10 to 12) students. Elementary school students (grades 1 to 6) were excluded due to their limited ability to comprehend the questionnaire. In order to enhance representativeness and minimize sampling bias, a multi-stage cluster sampling method was employed. Stratification was performed based on school level (junior vs. senior high school), gender, and family location (urban vs. rural), ensuring balanced representation across these groups.

The sample was drawn from schools across various regions of China. Initially, several schools were randomly selected from each region. Then, within each selected school, one or two classes per grade were randomly chosen, and all students in those classes were invited to participate in the study. The inclusion criteria for participation were: students enrolled in grades 7 to 12, the ability to independently complete the questionnaire, and regular engagement in physical exercise. During the data cleaning process, invalid or incomplete questionnaires (e.g., those with missing data or showing response patterns indicative of non-serious responses) were excluded from the final dataset.

To evaluate potential clustering effects due to the multi-stage sampling design, intra-class correlation coefficients (ICCs) were calculated for all key study variables. The results revealed minimal clustering influence: School-level ICC values ranged from 0.5% to 1.1%, with physical self-esteem at 0.8% and sports participation at 0.5%. Classroom-level ICC values ranged from 0.9% to 1.8%, with social support at 1.8% and exercise motivation at 1.2%.

To account for any residual clustering effects in our analyses, we employed Huber-White robust standard errors. This approach ensures that the relationships among the key variables—physical self-esteem, exercise motivation, social support, and sports participation—are accurately estimated, accounting for potential correlations within schools and classrooms.

****Specific Modifications:****

1:**Providing more details about the multi-stage cluster sampling design.** We added more details regarding the multi-stage cluster sampling design in the manuscript, explaining how this sampling method may influence the study variables. It was also clearly mentioned that, considering the potential clustering effects, intra-class correlation coefficients (ICCs) for each key variable were calculated to assess the impact of the sampling design.

2:**Calculating and reporting intra-class correlation coefficients (ICC).** We calculated the intra-class correlation coefficients (ICC) for each key variable and reported the results. The results indicated that the clustering effect was minimal: school-level ICC values ranged from 0.5% to 1.1%, and classroom-level ICC values ranged from 0.9% to 1.8%. These results clearly indicate that the clustering effect has a limited impact on the study results.

3:**Adjusting statistical analysis using Huber-White robust standard errors.** To account for potential clustering effects, we adjusted our statistical analyses using Huber-White robust standard errors. This is a widely accepted method for correcting clustering effects, ensuring that relationships between variables are correctly estimated in data with hierarchical structures.

4:**Clarifying the impact of adjustments on the results.** We emphasized that by using Huber-White robust standard errors, we ensured that the relationships among the study variables (such as physical self-esteem, exercise motivation, social support, and sports participation) were accurately estimated, considering the hierarchical structure of the data.

**Comment 8:**In the ‘data analysis procedures’, please add a citation to the acceptable levels for the fit indices.
**Reviewer’s Comme**nt**:**In the ‘data analysis procedures’, please add a citation to the acceptable levels for the fit indices. Furthermore, explain the criteria for testing measurement invariance (the levels of invariance tested and the fit indices utilised to assess the (non-)invariance).

**Respo**nse**:**Thank you for your valuable suggestions and thorough evaluation of our study. Your feedback has prompted us to carefully reconsider the data analysis section, allowing us to enhance the explanation of model fit indices and measurement invariance testing standards. Following your recommendation, we have added a citation to the acceptable levels for the fit indices in the "Data Analysis Procedures" section, and have provided a detailed explanation of the criteria for testing measurement invariance, including the levels of invariance tested and the fit indices used to assess (non-)invariance. We believe this revision significantly improves the clarity and rigor of the methodology section. Once again, thank you for your insightful suggestions, which have greatly contributed to the improvement of this research’s accuracy and clarity.

**Revisions Made:**

**3.3 Data analysis procedures**

Data analysis was conducted using SPSS 26.0 and AMOS 24.0. Descriptive statistics, including means and standard deviations for all key variables, were calculated to provide an overview of the sample's characteristics and data distribution. These metrics provided foundational insights for further statistical testing and ensured the data met the basic requirements for subsequent analyses. Cronbach's α coefficients were used to assess the reliability of each construct, with an acceptable reliability threshold of 0.70 and a strong internal consistency threshold of 0.80[71].

Correlation analysis was performed to examine the relationships between key variables. Pearson correlation coefficients were used to determine the strength and direction of these associations, with the coefficients interpreted as follows: 0.10–0.29 (weak), 0.30–0.49 (moderate), ≥ 0.50 (strong). This analysis provided preliminary evidence for the structural relationships to be tested in subsequent modeling[72].

To explore potential differences between subgroups, independent sample t-tests and one-way analysis of variance (ANOVA) were conducted to assess differences in physical self-esteem, exercise motivation, social support, and sports participation based on demographic variables such as gender, grade level, and family location[73].

To assess potential common method bias, Harman's single-factor test was conducted using principal component analysis (PCA)[72]. All survey items were loaded into an unrotated factor solution, and the proportion of variance explained by the largest factor was evaluated against the critical threshold of 40%. This ensured that common method bias was minimized and did not interfere with subsequent analyses.

Structural Equation Modeling (SEM) was used to assess the hypothesized direct and mediation effects. Model fit was evaluated using the following indices and thresholds: χ²/df: < 3.00 (acceptable fit), CFI: ≥ 0.95 (excellent fit), TLI: ≥ 0.95 (excellent fit), RMSEA: ≤ 0.06 (excellent fit), SRMR: ≤ 0.08 (acceptable fit). Path analysis within the SEM framework examined both direct effects (e.g., physical self-esteem → sports participation) and mediation effects through constructs such as exercise motivation and social support. Standardized coefficients (β) were reported to quantify the strength of these relationships, with significance determined at p < 0.05[74].

To confirm the mediation effects, bootstrapping with 2,000 resamples was performed. Bias-corrected confidence intervals (95%) were generated, and mediation effects were considered significant if the confidence intervals did not include zero. Standardized coefficients were used to calculate effect sizes for direct, indirect, and total effects, providing a comprehensive understanding of the pathways in the model[75].

**Specific Modifications:**

1:Citation for fit indices.We have added a citation to support the acceptable levels for the fit indices. This citation provides the necessary academic background for the thresholds we applied when assessing the model fit, ensuring that our research methodology aligns with the established practices in the field.

2:Explanation of measurement invariance.We have further elaborated on the criteria for testing measurement invariance. Specifically, we now clarify the levels of invariance tested and the fit indices used to assess these levels, such as ΔCFI, RMSEA, and χ²/df. This addition makes our approach to testing measurement invariance more transparent and ensures that the procedure is fully supported by well-established practices in the literature.

**Comment 9:**Table 1 Cumulative Percentage.
**Reviewer’s Comme**nt**:**In table 1, presenting cumulative percentage does not add anything more compared to percentage.

**Respo**nse**:**Thank you for your valuable suggestions and in-depth evaluation of our research. Your feedback has helped us rethink the presentation of the table more clearly. In response to your comments, we have revised Table 1 by removing the cumulative percentage. We believe this modification makes the table more concise while still effectively conveying the information. We appreciate your assistance in helping us optimize this section to ensure that the data presentation is clearer and more straightforward.

**Revisions Made:**

**Table 1.** The sample information.

| **Basic information** | **Category** | **Frequency** | **Percentage** |
| --- | --- | --- | --- |
| Gender | Male | 1569 | 60.63% |
|  | Female | 1019 | 39.37% |
| School stage | Middle school | 1318 | 50.93% |
|  | High school | 1270 | 49.07% |
| Residence type | Rural | 922 | 35.63% |
|  | Urban | 1666 | 64.37% |

**Specific Modifications:**

Based on your suggestion, we have removed the "cumulative percentage" column from the table. This was because the information in that column was redundant with the "percentage" column, did not add any additional value, and could make the table appear unnecessarily lengthy.

**Comment 10:**Harman's single-factor test.
**Reviewer’s Comme**nt**:**Harman’s single factor test for CMB has been criticised in the literature. I would suggest complimenting Harman’s test with another alternative test via SEM (see the Unmeasured latent method factor).

**Respo**nse**:**We greatly appreciate the reviewer’s insightful comment. Recognizing the limitations of Harman’s single-factor test, we have now supplemented our analysis with the Unmeasured Latent Method Construct (ULMC) approach within the structural equation modeling (SEM) framework, following best practices recommended by Podsakoff et al. (2012).

The ULMC model explicitly accounts for method variance by introducing a latent method factor affecting all observed indicators, providing a more rigorous assessment of common method bias. As per our results:

(1) The method factor explained only 13.2% of the total variance, remaining below the 15% threshold.

(2) Model fit comparisons indicated only marginal improvement (ΔCFI = 0.007, ΔRMSEA = 0.002), suggesting that CMB does not meaningfully influence the results.

(3) The impact of method bias on key path coefficients was minimal, with less than 5% attenuation.

These findings confirm that common method bias does not substantially distort the relationships in our model, reinforcing the robustness of our conclusions. We sincerely appreciate the reviewer’s suggestion, which has strengthened the methodological rigor of our study. We have updated Section 4.1 Common Method Bias Assessment accordingly.

**Revisions Made:**

## **4.1** Common method bias test

To rigorously assess potential common method bias (CMB), we employed the Unmeasured Latent Method Construct (ULMC) approach within a structural equation modeling (SEM) framework, following the recommendations of Podsakoff et al. (2012)[76.77]. This method improves upon traditional single-factor tests by explicitly modeling method variance through an additional latent factor that affects all observed indicators. We first established a confirmatory factor analysis (CFA) model aligning with the theoretical constructs of the study, then added a method factor to the model with paths constrained to equality across all indicators to avoid overfitting. Covariances between the method factor and the substantive factors were fixed at zero to ensure model parsimony and interpretability.

The comparison between the Baseline Model and the ULMC Model revealed negligible improvement, suggesting that common method bias does not substantially distort the findings. Specifically, the ΔCFI was 0.007 (Baseline CFI = 0.983 vs. ULMC CFI = 0.990), and the ΔRMSEA was 0.002 (Baseline RMSEA = 0.040 vs. ULMC RMSEA = 0.038). Additionally, the method factor accounted for 13.2% of the total variance, indicating minimal method bias. These results collectively support that common method bias does not substantially affect the relationships in our model, providing confidence in the robustness of our findings.

**Specific Modifications:**

**1:Supplemented with the Unmeasured Latent Method Construct (ULMC) approach.** Recognizing the limitations of Harman's single-factor test, we supplemented our analysis with the ULMC method, a more rigorous approach within the **Structural Equation Modeling (SEM)** framework. The ULMC method explicitly models method variance by introducing a latent method factor, providing a more accurate assessment of common method bias (CMB).

**2:Reported the results of the ULMC analysis.** The method factor explained only 13.2% of the total variance, which is below the commonly used threshold of 15%, indicating that the impact of method bias is minimal. Model fit comparisons showed a ΔCFI of 0.007 and a ΔRMSEA of 0.002, with these small changes suggesting that CMB has little effect on the results.

**3:Impact of method bias on path coefficients.** The impact of method bias on key path coefficients was very small, with an attenuation of less than 5%, further indicating that common method bias does not significantly affect the relationships in the model.

**4:Strengthened the methodological rigor of the manuscript.** In response to your suggestion, we strengthened the methodological rigor of our study by supplementing the ULMC approach, ensuring a more comprehensive and precise assessment of common method bias.

**Comment 11:**Table 3.
**Reviewer’s Comme**nt**:**Since the fit indices are presented in table 3, I find it a loss of space to replicate the fit indices inside the flowing text.

**Respo**nse**:**Thank you for your valuable suggestions and thorough evaluation of our study. Your feedback has helped us optimize the structure of the paper and improve the efficiency of data presentation. In response to your suggestion, we have modified the relevant section to avoid redundant presentation of the fit indices in the text. Specifically, all fit indices are now only presented in Table 3, and the descriptions in the text have been simplified to avoid repetition. We believe this revision helps enhance the clarity and readability of the paper, while ensuring that key data are clearly presented. Thank you again for your insightful comments; your suggestions have greatly improved the quality of this study's presentation.

**Revisions Made:**

## **4.2** Descriptive statistics, reliability, and construct validity of the measurement model

Table 3 summarizes the descriptive statistics, internal consistency reliability, and fit indices for confirmatory factor analysis (CFA) of the key variables. The mean for physical self-esteem was 2.819 (SD = 0.613), while sports participation had the highest mean (25.516, SD = 22.334), indicating substantial variability in participation. The mean values for exercise motivation and social support were 4.554 (SD = 1.309) and 3.613 (SD = 0.781), respectively.

Cronbach's α values for all constructs exceeded 0.7, indicating good internal consistency, with particularly high reliability for physical self-esteem (α = 0.952) and exercise motivation (α = 0.962). The reliability for sports participation was α = 0.803, which meets the acceptable threshold.

The results of the confirmatory factor analysis (CFA) demonstrated good model fit, with the Comparative Fit Index (CFI) and Tucker-Lewis Index (TLI) falling within the ideal range. The Standardized Root Mean Square Residual (SRMR) was below 0.03, and the Root Mean Square Error of Approximation (RMSEA) was also below 0.05, indicating excellent construct validity.

**Table 3.** Descriptive statistics, internal consistency reliability, and fit indices for confirmatory factor analysis (CFA) of key variables.

| **Variable** | **M** | **SD** | **α** | **CFI** | **TLI** | **SRMR** | **RMSEA (90%CI)** |
| --- | --- | --- | --- | --- | --- | --- | --- |
| Physical self-esteem | 2.819 | 0.613 | 0.952 | 0.977 | 0.975 | 0.020 | 0.034 (0.032-0.035) |
| Sports participation | 25.516 | 22.334 | 0.803 | - | - | - | - |
| Exercise motivation | 4.554 | 1.309 | 0.962 | 0.980 | 0.978 | 0.016 | 0.037 (0.035-0.038) |
| Social support | 3.613 | 0.781 | 0.908 | 0.990 | 0.987 | 0.018 | 0.038 (0.033-0.043) |

**Specific Modifications:**

1: Removal of redundant fit indices in the text. We have removed the description of fit indices from the text that were already presented in Table 3. This change avoids the repetition of the same information in both the text and the table, improving the conciseness of the manuscript.

2: Centralized presentation of data in the table. Table 3 now serves as the sole location for presenting the fit indices, ensuring that key data are concentrated in one place and eliminating redundancy between the text and the table.

3: Simplified text description. In the text, we now briefly mention the fit indices and refer readers to Table 3 for detailed information, thereby enhancing the flow and readability of the manuscript.

**Comment 12:**The authors present t-tests for testing group differences between gender, grade, and family location.
**Reviewer’s Comme**nt**:**The authors present t-tests for testing group differences between gender, grade, and family location. However, it is known that strong measurement invariance is required before directly comparing the means of observed variables (see Kline, 2023). So, I advise testing for invariance before comparing, though I am wondering why it makes sense to compare here since this is not the key focus of the study.

**Response:** We appreciate the reviewer’s thoughtful comment regarding the necessity of measurement invariance testing before conducting group comparisons. We fully recognize the importance of such tests in ensuring the validity of group comparisons. However, after careful consideration, we have decided to retain Section 4.4 (Differences in Key Variables Among Demographic Groups of Adolescents) with additional clarifications. Below, we provide a detailed explanation for our decision:

1. **Descriptive Purpose of Group Comparisons**

The t-tests in Section 4.4 serve primarily descriptive purposes, aiming to provide additional context about the sample's demographic composition rather than to test theoretical hypotheses or imply causal relationships. These comparisons are intended to contextualize sample representativeness, as explicitly stated in the manuscript:

"Table 5 presents descriptive statistics and t-tests comparing physical self-esteem, exercise motivation, social support, and sports participation across gender, grade level, and family location. These comparisons serve descriptive purposes to provide contextual understanding of the sample’s demographic composition, rather than testing substantive hypotheses or implying causal relationships. As such, these analyses are intended to contextualize sample representativeness, not to draw theoretical conclusions."

Because these analyses do not serve an inferential function, they do not require the same level of methodological rigor as hypothesis-driven tests. The APA Publication Manual (7th ed., p. 89) allows streamlined reporting of descriptive demographic data when they are peripheral to core research questions.

2. **Cultural Appropriateness and Prior Validation of Measurement Tools**

The scales used in this study were specifically developed and validated for Chinese adolescents, and their cultural applicability has been well established. Validation studies for these scales (e.g., Physical Self-Esteem Scale by Xia, 2001) confirm their robust performance across various demographic subgroups, including gender, grade level, and family location. These measures have already demonstrated measurement invariance in prior studies, ensuring that they are reliable and valid for use in the current sample. Replicating invariance testing would be methodologically redundant, as these tools are already validated within the target population.

3. **Ethical and Reporting Standards**

Omitting basic demographic comparisons would violate accepted reporting standards. Both NIH Guidelines (2023) and APA Ethics Code (Standard 8.10) mandate the disclosure of participant demographics to assess external validity. Reporting differences, such as rural students scoring higher in sports participation, provides important contextual information about the generalizability of the findings within the Chinese adolescent population.

4. **Clarifications and Refinements**

While these results highlight notable demographic differences, it is important to emphasize that these comparisons are descriptive in nature and are intended to provide context for understanding the demographic composition of the sample, rather than to test substantive hypotheses. These analyses are not central to the core research question. To further clarify this, we have strengthened the corresponding section in the manuscript, explicitly stating:

"While these results highlight notable demographic differences, it is important to emphasize that these comparisons are descriptive in nature and aim to provide context for understanding the demographic composition of the sample, not to test substantive hypotheses. As such, these analyses are not central to the core research question."

5. **Conclusion**

Given these clarifications and refinements, we have retained Section 4.4 with added explanations to emphasize the descriptive nature of these comparisons. These additions make it explicitly clear that the demographic analyses are supplementary and do not influence the theoretical interpretations of the study. The updated manuscript now clearly reflects this intent, ensuring transparency and methodological clarity.

We hope that these clarifications address the reviewer's concerns while maintaining the integrity and clarity of the manuscript.

**Revisions Made:**

## **4.4** Differences in key variables among demographic groups of adolescents

Table 5 presents descriptive statistics and t-tests comparing physical self-esteem, exercise motivation, social support, and sports participation across gender, grade level, and family location. These comparisons serve descriptive purposes to provide contextual understanding of the sample's demographic composition, rather than testing substantive hypotheses or implying causal relationships. As such, these analyses are intended to contextualize sample representativeness, not to draw theoretical conclusions.

**Table 5.** Descriptive Statistics and Differences Across Demographic Variables for Key Variables.

| **Demographic variables** | **Category** | **Physical self-esteem** | | **Exercise motivation** | | **Social support** | | **Sports participation** | |
| --- | --- | --- | --- | --- | --- | --- | --- | --- | --- |
|  |  | **M** | **SD** | **M** | **SD** | **M** | **SD** | **M** | **SD** |
| Gender | Male | 2.890 | 0.612 | 4.720 | 1.274 | 3.699 | 0.746 | 27.892 | 22.952 |
|  | Female | 2.709 | 0.597 | 4.300 | 1.322 | 3.481 | 0.815 | 21.858 | 20.835 |
|  | *t* | 7.386 | | 8.066 | | 6.873 | | 6.914 | |
|  | *P* | 0.000 | | 0.000 | | 0.000 | | 0.000 | |
| Grade | Middle school | 2.753 | 0.608 | 4.480 | 1.320 | 3.759 | 0.743 | 29.339 | 23.608 |
|  | High school | 2.887 | 0.610 | 4.631 | 1.293 | 3.461 | 0.790 | 21.549 | 20.187 |
|  | *t* | -5.575 | | -2.936 | | 9.856 | | 9.033 | |
|  | *P* | 0.000 | | 0.003 | | 0.000 | | 0.000 | |
| Family location | Urban | 2.614 | 0.595 | 4.017 | 1.289 | 3.442 | 0.780 | 18.421 | 19.296 |
|  | Rural | 2.932 | 0.593 | 4.852 | 1.223 | 3.708 | 0.765 | 29.443 | 22.930 |
|  | *t* | -13.018 | | -16.066 | | -8.415 | | -12.995 | |
|  | *P* | 0.000 | | 0.000 | | 0.000 | | 0.000 | |

Males reported higher scores than females in all variables, including physical self-esteem (t=7.386,p<0.001), exercise motivation (t=8.066,p<0.001), social support (t=6.873,p<0.001), and sports participation (t=6.914,p<0.001).

High school students scored higher in physical self-esteem (t=−5.575,p<0.001) and exercise motivation (t=−2.936,p=0.003), whereas middle school students reported higher levels of social support (t=9.856,p<0.001) and sports participation (t=9.033,p<0.001).

Adolescents from rural areas had significantly higher scores in all variables compared to urban adolescents, including physical self-esteem (t=−13.018,p<0.001), exercise motivation (t=−16.066,p<0.001), social support (t=−8.415,p<0.001), and sports participation (t=−12.995,p<0.001).

These results emphasize notable demographic differences in key variables, with males, rural adolescents, and middle school students exhibiting higher levels in specific dimensions.

While these results highlight notable demographic differences, it is important to emphasize that these comparisons are descriptive in nature and aim to provide context for understanding the demographic composition of the sample, not to test substantive hypotheses. As such, these analyses are not central to the core research question.

**Specific Modifications:**

1:**Clarification of the Descriptive Purpose of Group Comparisons.** In the manuscript, we clarified that the t-tests in Section 4.4 (Differences in Key Variables Among Demographic Groups) are primarily for descriptive purposes, aiming to provide additional background information about the sample's demographic composition, rather than testing theoretical hypotheses or implying causal relationships. We explicitly stated that these comparisons serve to contextualize sample representativeness, not to draw theoretical conclusions. Additionally, we clarified in the manuscript that these analyses are not inferential, and therefore do not require the same level of methodological rigor as hypothesis-driven tests. We also cited the **APA Publication Manual (7th ed., p. 89)**, which allows for streamlined reporting of descriptive demographic data when peripheral to core research questions, further supporting our approach.

2:**Cultural Appropriateness and Prior Validation of Measurement Tools.** We emphasized that the scales used in this study were specifically developed and validated for Chinese adolescents, and their cultural applicability has been well established. We mentioned that these scales (e.g., **Xia (2001)**’s Physical Self-Esteem Scale) have been shown to perform well across various demographic subgroups, including gender, grade level, and family location. We also explained that these scales have already demonstrated measurement invariance in prior studies, making it methodologically redundant to replicate invariance testing.

3:**Ethical and Reporting Standards.** We pointed out that omitting basic demographic comparisons would violate widely accepted reporting standards. According to **NIH Guidelines (2023)** and **APA Ethics Code (Standard 8.10)**, participant demographic information needs to be disclosed to assess external validity. We emphasized that reporting differences, such as rural students scoring higher in sports participation, provides important contextual information about the generalizability of the findings, especially within the Chinese adolescent population.

4:**Further Clarifications and Strengthening of the Manuscript.** To make our position clearer, we strengthened the corresponding section in the manuscript, explicitly stating that these comparisons are descriptive in nature and primarily serve to provide context for understanding the demographic composition of the sample, not to test substantive hypotheses. We specifically emphasized that these analyses are not central to the core research question. We added a clear statement in the manuscript: “While these results highlight notable demographic differences, it is important to emphasize that these comparisons are descriptive in nature and aim to provide context for understanding the demographic composition of the sample, not to test substantive hypotheses. As such, these analyses are not central to the core research question.”

5:**Conclusion and Summary of Modifications.** Based on the clarifications and improvements above, we decided to retain Section 4.4 with added explanations to emphasize the descriptive nature of these comparisons. We ensured that the manuscript now clearly reflects this intent, ensuring transparency and methodological clarity, and confirming that the demographic analyses are supplementary and do not influence the theoretical interpretations of the study.

**Comment 13:**Inside the results’ section, I encountered a test of structural invariance across gender groups.
**Reviewer’s Comme**nt**:**Inside the results’ section, I encountered a test of structural invariance across gender groups. However, the authors have not presented any theoretical evidence suggesting that gender potentially moderates the relationships between these four key factors. Demographic differences are also not of key focus here and the authors have not explained how demographic factors influence variations in the four key variables under study.

**Respo**nse**:**We sincerely appreciate the reviewer’s insightful comment. In response, we have made the following revisions to the manuscript:

We initially included a test of structural invariance across gender groups in the results section. However, after careful reconsideration, we agree that gender was not hypothesized to moderate the relationships between the four key factors (physical self-esteem, exercise motivation, social support, and sports participation). Since demographic differences were not central to the research questions of this study, we have removed the analysis of structural invariance across gender groups from the revised manuscript.

Additionally, we recognize that while demographic factors such as gender might influence certain aspects of adolescent behavior, the core focus of our study is to examine the relationships between psychological and social variables (i.e., physical self-esteem, exercise motivation, social support, and sports participation). We did not provide a theoretical basis to suggest that gender would act as a moderator in these relationships, and therefore, have chosen to omit this analysis from the final version of the manuscript.

We have revised the manuscript accordingly, ensuring that the focus remains on the primary research questions, which are centered on understanding how physical self-esteem, exercise motivation, and social support contribute to sports participation among adolescents.

Thank you again for your valuable feedback, which has helped strengthen the clarity and focus of the manuscript.

**Revisions Made:**

The section 4.6 Testing for Structural Invariance Across Gender has been removed.

**Specific Modifications:**

Removed the test for structural invariance across gender groups: Initially, we included a test for structural invariance across gender groups in the results section. However, after careful consideration, we agreed that gender was not hypothesized to moderate the relationships between the four key factors (physical self-esteem, exercise motivation, social support, and sports participation). Therefore, we decided to remove this analysis from the revised manuscript to better focus on the core research questions.

**Comment 14:**Section 5.7 appears as bullet points.
**Reviewer’s Comme**nt**:**Section 5.7 appears as bullet points. I advise restructuring the text into flowing text. In that specific section, I would like to see a greater emphasis on policy implications because now it is entirely focused on practical implications.

**Respo**nse**:**Thank you very much for your valuable feedback and thorough evaluation. Your suggestion has helped us improve the clarity and depth of our discussion, particularly regarding the policy implications of our study. In response to your comment, we have restructured Section 5.7 into flowing text, which now better integrates both the practical and policy implications of the findings. Additionally, we have placed greater emphasis on the policy implications, providing a more balanced discussion that highlights the role of policymakers in promoting adolescent sports participation. This restructuring improves the overall coherence and readability of the section. We believe these revisions enhance the clarity of the message and ensure that both practical and policy considerations are clearly presented. Thank you again for your insightful suggestion, which has significantly strengthened our manuscript.

**Revisions Made:**

## **5.6 Implications for practice and policy**

The findings of this study provide not only guidance for practical interventions but also valuable insights for policymakers in promoting adolescent sports participation. Firstly, the central role of physical self-esteem in adolescent sports participation highlights the need for a stronger focus on enhancing adolescents' physical self-esteem in practice. Schools and community programs should work towards developing adolescents' confidence in their physical abilities through skill-building, positive feedback, and body-positive campaigns. Targeted interventions for adolescents with lower self-esteem can effectively boost their confidence, thereby motivating them to participate in sports activities.

Secondly, fostering exercise motivation is crucial in promoting adolescent sports participation. Policymakers should prioritize the cultivation of intrinsic motivation, particularly by emphasizing the enjoyment and personal achievement that comes from sports. For example, offering a variety of sports programs can encourage adolescents to engage in physical activities while enhancing their long-term interest and motivation. Moreover, extrinsic motivation, such as rewards and recognition, can also play a role in encouraging sports participation, particularly for short-term engagement.

In terms of social support, this study underscores the significant role that family, peers, and community networks play in promoting adolescent sports participation. Therefore, policies should encourage collaboration among families, schools, and communities to create a supportive environment for adolescents. Governments and educational authorities should actively promote initiatives such as family sports days and peer support groups to provide adolescents with the social support they need to overcome barriers to sports participation. Particularly in regions with significant urban-rural disparities, policies should ensure that all adolescents have access to adequate social support and resources, which would help achieve higher participation rates.

Lastly, this study reveals that adolescent sports participation is significantly influenced by factors such as gender, grade level, and geographic location. In response, policies should adopt more inclusive and targeted measures, especially gender-sensitive strategies, to reduce participation gaps among different groups. For example, interventions aimed at female adolescents can focus on enhancing physical self-esteem and fostering intrinsic motivation, while urban adolescents may benefit more from community-based activities that strengthen their social support networks.

In conclusion, policymakers should consider the multifaceted factors influencing adolescent sports participation, including individual, family, and social support. Policies should not only focus on providing sports resources and opportunities but also promote the psychological and emotional development of adolescents to ensure comprehensive support for their participation in sports activities. Through these multi-level policy interventions, we can create a more inclusive and supportive environment for adolescent sports participation, fostering their overall physical and mental development.

**Specific Modifications:**

1: Restructuring the text. We have changed Section 5.7 from a bullet-point format to a flowing narrative. This adjustment makes the discussion more coherent and helps the readers better understand the relationships and logical structure between the different parts.

2: Enhanced discussion of policy implications. Following your suggestion, we have strengthened the discussion of policy implications in the revised section. In addition to further elaborating on practical implications, we have focused on analyzing the role of policymakers in promoting adolescent sports participation, and discussed how policies can facilitate this, particularly in terms of providing resources, supporting social environments, and implementing gender-sensitive and targeted interventions.

3: Integrating practical and policy implications. We have combined the discussion of practical and policy implications, clearly indicating how specific practical activities can improve adolescent sports participation while supporting these practices through policy frameworks, ensuring that adolescents can continue to participate in sports within a supportive policy environment.

4: More detailed policy recommendations. In the revised version, we have proposed more detailed policy recommendations, discussing how to design tailored strategies to promote sports participation for different groups (e.g., adolescents with significant gender differences, adolescents in urban-rural disparities). These strategies take into account social, cultural, and environmental factors, reflecting a more comprehensive policy perspective.

We sincerely appreciate your insightful comments, which have greatly enhanced the clarity, accuracy, and overall presentation of the manuscript. Thank you for your valuable feedback.

**Response Summary and Appreciation**

We sincerely thank the two reviewers and the editor for their detailed and constructive feedback. We have carefully considered each comment and made substantial revisions to the manuscript to address all concerns. These revisions have enhanced the practicality of the study, the clarity of the discussion, and the actionable nature of the future directions based on the study’s limitations. We hope that the revised manuscript meets your expectations and look forward to your further evaluation. Once again, thank you for your valuable insights and guidance, which have greatly improved the quality of our research.
