# Supplementary material for: From physical self-esteem to sports participation: The mediating role of exercise motivation and social support in adolescents
Source: PLoS One. 2025 May 22;20(5):e0324588. doi: 10.1371/journal.pone.0324588 (PMC12097568; doi:10.1371/journal.pone.0324588)
Supplement: Attachment — Submitted filename: Response_to_Reviewers_auresp_2.docx [file pone.0324588.s004.docx]

Response to Reviewers

We would like to express our sincere gratitude to all three reviewers for their thorough and constructive comments on our manuscript. Your insightful feedback has been invaluable in helping us improve the quality, rigor, and clarity of our manuscript. We carefully reviewed all the comments and have made substantial revisions to address the concerns raised. Below, we provide a point-by-point response to each of the reviewers’ comments, detailing the revisions we have made.

Once again, we deeply appreciate your time, expertise, and constructive suggestions, which have significantly strengthened our study. We hope that the revised manuscript meets your expectations.

**Reviewer 1**

**Comment 1:**I reccomend the manuscript to be published in PLOS ONE.
**Reviewer’s Comme**nt**:**The Authors adressed all recommended amendments. I reccomend the manuscript to be published in PLOS ONE.

**Respo**nse**:**Thank you to the reviewer for recognizing and supporting our manuscript. We greatly appreciate your acknowledgment of the revisions we made and for recommending our manuscript for publication in PLOS ONE. Your valuable feedback and guidance have played a crucial role in improving the quality of our research.

**Reviewer 3**

**Comment 1:**The recognition of the research.
**Reviewer’s Comme**nt**:** I recently had the opportunity to read Author´s manuscript. It shows a noteworthy study with sophisticated analysis. I would like to highlight that the Authors dedicated time and effort to address all comments made by the previous Reviewers. I compared the original submission to the revised one and, the modifications improved substantially the structure and the content of the manuscript. I appreciate the endeavor and creativity the Authors invested in this work.

**Respo**nse**:**Thank you to the reviewer for the recognition and appreciation of our manuscript. We greatly appreciate your acknowledgment of the time and effort we invested in addressing the comments from previous reviewers. Your praise is highly meaningful to us and motivates us to continue improving the quality of our research. Thank you for recognizing the creativity and effort we put into this work.

**Comment 2:**Figure 1.
**Reviewer’s Comme**nt**:** Figure 1 is mentioned in Theoretical Model, however the Figure 1 per se is not available throughout the document.

**Respo**nse**:**Thank you for your thoughtful feedback. We have noted that Figure 1 is mentioned in the "Theoretical Model" but was not included in the document. We have made the necessary revisions to ensure that Figure 1 is properly presented in the manuscript. Thank you for your valuable suggestion.

**Revisions Made:**

****Figure 1.**** Theoretical model of the impact of adolescent p**hysical self-esteem** on s**ports participation**: the chain mediation effect of e**xercise motivation** and s**ocial support**.

**Specific Modifications:**

Thank you for your feedback. In response to your comment about Figure 1 being mentioned in the "Theoretical Model" but not presented in the document, we have now inserted Figure 1 into the manuscript to ensure it is properly displayed in the relevant section. Thank you for your valuable suggestion.

**Comment 3:**Reformulate the statement of Hypothesis H4.
**Reviewer’s Comme**nt**:**I would word the Hypothesis4(H4) to emphasize the joint mediation that occurs with exercise motivation and social support: “Exercise motivation and social support jointly mediate the relationship between physical self-esteem and sports participation in adolescents.” For reference: VanderWeele TJ, Vansteelandt S. Mediation Analysis with Multiple Mediators. Epidemiol Methods. 2014 Jan;2(1):95-115. doi: 10.1515/em-2012-0010. PMID: 25580377; PMCID: PMC4287269.

**Respo**nse**:**Thank you for your valuable suggestion. We have revised Hypothesis H4 based on your feedback to emphasize the joint mediation of exercise motivation and social support. The revised statement is: "Exercise motivation and social support jointly mediate the relationship between physical self-esteem and sports participation in adolescents." Thank you for your careful guidance, which has helped us express the hypothesis more clearly.

**Revisions Made:**

**Hypothesis4(H4): Exercise motivation and social support jointly mediate the relationship between physical self-esteem and sports participation in adolescents.**

**Specific Modifications:**

Thank you for your suggestion. Based on your feedback, we have revised the statement of Hypothesis H4 to better emphasize the joint mediation of exercise motivation and social support. The revised statement is: "Exercise motivation and social support jointly mediate the relationship between physical self-esteem and sports participation in adolescents." This modification makes the hypothesis clearer and aligns with your suggestion. Thank you for your careful guidance.

**Comment 4:** I suggest including the new Table 8 to the manuscript, or removing the sentence.
**Reviewer’s Comme**nt**:** I understood that Table 8 “Testing for structural invariance across gender” was removed to address previous Reviewer’s request. Yet, the following sentence was found: “The mediation analysis should be summarized in Table 8.” I suggest including the new Table 8 to the manuscript, or removing the sentence.

**Respo**nse**:**Thank you for your valuable feedback. We noticed that during the revision process, we mistakenly labeled Table 7 as Table 8. In response to your suggestion, we have made the necessary corrections and revised the inaccurate sentence. Thank you for your careful guidance, which has helped us improve the accuracy of the manuscript.

**Revisions Made:**

The mediation analysis results are summarized in Table 7:

**Specific Modifications:**

Thank you for your suggestion. During the revision process, we indeed removed Table 8 "Testing for structural invariance across gender" to address the previous reviewer's request. We noticed that, during the revision, we mistakenly labeled Table 7 as Table 8. In response to your suggestion, we have made the necessary corrections and revised the inaccurate sentence to ensure consistency and accuracy in the manuscript. Thank you for your careful guidance.

**Comment 5:** The box issue in Table 2.

**Reviewer’s Comme**nt**:** I noticed that the Authors corrected the squares that appeared in Table 2, as previous recommendations. However, parenthesis observed in Liwei Zhang (2004), found in row “Exercise motivation”, column “Author (Year)”, seem different from the others (perhaps a different font? Size? Space?).

**Respo**nse**:**Thank you for your detailed feedback. We noticed that the parentheses in Liwei Zhang (2004) in the "Exercise motivation" row and the "Author (Year)" column of Table 2 were different from the others. We have made the necessary corrections to standardize the font, size, and spacing of the parentheses to ensure consistency in the table. Thank you for your valuable suggestion.

**Revisions Made:**

**Table 2.** Scales used in this study.

| **Scale** | **Author (Year)** | **Item quantity** | **Scoring** | **Dimensions** |
| --- | --- | --- | --- | --- |
| Physical self-esteem | **Xia Xu(2001)** | 30 | 4 | **Physical self-worth;**  **Sport ability;**  **Physical condition;**  **Physical attractiveness;**  **Physical fitness** |
| Physical exercise | Deqing Liang (1994) | 3 | 5 | Exercise intensit; Duratio;  exercise Frequency |
| Exercise motivation | Liwei Zhang  (2004) | 28 | 7 | **Intrinsic motivation;**  **Extrinsic motivation;**  **Amotivation** |
| Social support | Wei Chen(2016) | 12 | 5 | **Family;**  **Friends;**  **Others** |

**Specific Modifications:**

Thank you for your detailed feedback. We have noticed that the parentheses used for Liwei Zhang (2004) in the "Exercise motivation" row and the "Author (Year)" column of Table 2 were different from the others. To address this, we have standardized the font, size, and spacing of the parentheses to ensure consistency with the rest. Thank you for your valuable suggestion, which has helped improve the consistency of the table.

We sincerely appreciate your insightful comments, which have greatly enhanced the clarity, accuracy, and overall presentation of the manuscript. Thank you for your valuable feedback.

**Reviewer 4**

**Comment 1:** The moderating role of social support.

**Reviewer’s Comme**nt**:** From the manuscript as is currently written, I think the authors might want to investigate the following: the role of social support as a moderator, which would then be a moderated mediation analysis. Because social support is extrinsic to an individual, this is why it might be more of a moderator than a mediator. Social support is outside of the control of an individual, so it moderates the relation between your independent and dependent variable, along with the mediating role of exercise motivation.

**Respo**nse**:**Thank you very much for this thoughtful and theoretically grounded suggestion. We fully acknowledge that social support, as a contextual and externally sourced factor, is often conceptualized as a moderator in behavioral models, particularly when it is positioned as a buffer or amplifier of the relationship between personal traits and behavioral outcomes.

However, in the present study, we selected a mediating role for social support based on two key considerations:

Perceived social support—the construct used in this study—is not merely an objective external condition, but a subjective experience shaped by personal traits such as self-esteem, emotional openness, and interpersonal style. Prior studies have shown that individuals with higher physical self-esteem are more likely to perceive, seek, and internalize support from others. In this sense, perceived social support may function as a psychosocial response elicited by physical self-esteem rather than a purely exogenous moderator.

Our empirical results show that physical self-esteem significantly predicts perceived social support (β = 0.17, p < 0.001), which supports the logic of a causal path from self-esteem to support perception. Moreover, in our sequential mediation analysis, social support was found to partially transmit the effect of exercise motivation to sports participation, suggesting its functional position within an internal psychological chain rather than as a conditional boundary factor.

That said, we agree with you that an alternative conceptualization of social support as a moderator (e.g., moderating the path from motivation to behavior) is theoretically meaningful and offers a valuable avenue for future research. We have added a corresponding discussion in the Limitations and Future Directions section (Section 6), where we explicitly propose testing a moderated mediation model in future studies. This would allow for a more comprehensive examination of the boundary conditions under which physical self-esteem and motivation influence behavioral engagement.

We appreciate your input, which has helped us further reflect on the conceptual positioning of key variables and enriched the theoretical scope of the study.

**Comment 2:** Robustness testing.

**Reviewer’s Comme**nt**:**  Further, although in the study, it was found that the structure of the model was invariant across gender, additional testing for invariance should have been conducted for school stage and residence type.

**Respo**nse**:**We appreciate your thoughtful suggestion regarding additional invariance testing across school stage and residence type. Indeed, multi-group comparisons can provide valuable insights into the stability of structural relationships across subpopulations.

However, following a recommendation from another reviewer who expressed concerns about the manuscript's length and model complexity, we have removed all measurement invariance testing from the revised version to maintain focus and coherence. This decision was made to streamline the presentation and emphasize the core mediation model and its practical implications.

That said, we fully agree that testing for structural equivalence across demographic groups is a meaningful direction for future research. Accordingly, we have added a note in the Limitations and Future Directions section (Section 6) indicating that future studies should incorporate measurement invariance analyses across different demographic strata (e.g., school stage and residence type) to enhance generalizability and model robustness.

We sincerely appreciate your insightful comments, which have greatly enhanced the clarity, accuracy, and overall presentation of the manuscript. Thank you for your valuable feedback.

**Response Summary and Appreciation**

We sincerely thank the three reviewers and the editor for their detailed and constructive feedback. We have carefully considered each comment and made substantial revisions to the manuscript to address all concerns. These revisions have enhanced the practicality of the study, the clarity of the discussion, and the actionable nature of the future directions based on the study's limitations. We hope that the revised manuscript meets your expectations and look forward to your further evaluation. Once again, thank you for your valuable insights and guidance, which have greatly improved the quality of our research.
